# Reconstructing subsurface fracture geometries in rock slope instabilities through ambient vibration-based numerical modelling inversion

**DOI:** 10.1038/s41598-026-39538-9

**Published:** 2026-02-10

**Authors:** Guglielmo Grechi, Jeffrey R. Moore, Sebastiano D’Amico, Salvatore Martino

**Affiliations:** 1 Department of Earth Sciences, Sapienza University of Rome, P.le Aldo Moro 5, Rome, 00185 Italy; 2 CERI, Research Centre for Geological Risks, P.le Aldo Moro 5, Rome, 00185 Italy; 3 Department of Geology and Geophysics, University of Utah, 115 S 1460 E, Salt Lake City, UT 84112 USA; 4 Department of Geosciences, University of Malta, Msida, 2080 MSD Malta

**Keywords:** Ambient vibrations, Slope stability, Operational modal analysis, Numerical modelling, Engineering, Natural hazards, Solid Earth sciences

## Abstract

Detailed engineering-geological models are crucial for assessing landslide hazards, yet their reliability is limited when poorly defined fracture networks control slope failure mechanisms. Traditional surveying techniques often fail to accurately constrain fracture extents, resulting in oversimplified and uncertain boundary conditions. We address these limitations by integrating array-based ambient vibration modal analysis with numerical modelling to invert for the subsurface geometry of fracture-controlled rock slope instabilities. We applied our approach at two case studies exhibiting similar toppling failure mechanisms. Linear seismic arrays were deployed to record ambient vibrations and derive resonance frequencies and 3D mode shapes using the Frequency Domain Decomposition technique. We then constructed 3D finite-element models representing the unstable rock volumes with their rear boundaries segmented into regular grids to simulate thousands of unique fracture configurations. Model results were compared with field-derived modal parameters using a multi-metric similarity ranking score evaluating resonance frequency and mode shape consistency. Results revealed ensembles of top-performing models that reproduced the observed resonance modes and converged toward fracture geometries consistent with field-estimated fracture depths. Inversion stability increased with the number of resonance modes considered, highlighting the need for multiple constraints. Our results demonstrate that integrating ambient vibration field surveys with numerical modal analysis can support quantitative description of subsurface boundary conditions in unstable rock slopes, providing a robust framework for improved landslide structural characterization and monitoring.

## Introduction

Detailed geological models of unstable rock slopes are essential for accurate landslide hazard assessment, yet their accurate construction still represents an overarching challenge in engineering geology^1–3^. Geostructural models distill information on complex geometries, boundary conditions and material properties into simplified, schematic representations of geological structures by integrating results from field mapping, remote surveys, geotechnical and geophysical investigations^4–8^. Despite recent technological advances in surveying methods, developing representative subsurface models of rock slope instabilities is hindered by inherent observational limitations, particularly when discrete fractures control slope failure mechanisms^9^. Field surveying techniques, such as LiDAR and photogrammetry, can yield accurate data on surface conditions but cannot resolve fracture persistence at depth^10^. Similarly, boreholes can provide high-quality data, but they are invasive, costly, and offer low spatial resolution^11^. Near-surface geophysical investigations have also proved effective in locating rock mass fractures, although their resolution often remains insufficient to constrain detailed fracture geometry and persistence^12,13^.

In recent years, ambient vibration modal analysis has emerged as a promising technique for enhanced site characterization, monitoring, and hazard assessment of unstable rock slopes and freestanding natural landforms^2,14–19^. The dynamic properties of landforms (i.e., modal frequencies, mode shapes, and damping ratios) depend on their geometry, material properties, and boundary conditions^20,21^, thereby carrying relevant information for refining geostructural models^1,22–25^. Modal frequencies and mode shapes are particularly informative for characterizing slope instabilities and describing their internal structure, as they can integrate field observations to identify fracture-isolated compartments with unique dynamic properties. These parameters can also be monitored for structural health assessment, as drifts from baseline values indicate both reversible changes in material properties and permanent damage accumulation^18,19,26–29^. However, while modal frequencies are comparably easy to track over time, identifying variations in mode shapes is often difficult because they can reflect complex, localized changes in fracture stiffness and continuity within the rock mass^30^.

In this study, we hypothesize that modal parameters—specifically resonance frequencies and mode shapes—can be used to invert for subsurface boundary conditions in unstable rock slopes. Previous studies have successfully coupled experimental data with numerical modelling to constrain material properties and boundary conditions of rock slopes, natural arches and towers, and even trees or cacti^1,30–35^. In particular, Bessette-Kirton et al.^1^ demonstrated that numerical models calibrated on field data could replicate the observed dynamic behavior of a toppling rock slab by systematically varying the geometry and continuity of a rear fracture boundary. Their results emphasized the potential of combining field and numerical modal analyses to reconstruct geostructural features, aiding improved site characterization. However, their modelling setup relied on initial, deterministic geometric inputs derived from field measurements of fracture depth, thereby limiting the range of boundary conditions explored. Such constraints can bias interpretations of fracture persistence at depth, as field observations are often sparse and limited by access and spatial resolution. We therefore sought an analytical approach in which consistency in measured dynamic behavior drives the inversion process rather than deterministic geometric inputs. Building on our hypothesis, we developed a stochastic, rule-based modelling approach to explore a wide range of fracture configurations and identify models that best reproduce field data. Our workflow is independent of deterministic initial assumptions about fracture geometry (e.g., prescribed fracture persistence or spatial continuity), relying solely on agreement between field and numerical modal parameters to identify dynamically admissible configurations. Moreover, model performance is evaluated using a multi-metric ranking score to quantify consistency in simulated modal frequencies and mode shapes. This strategy enables data-driven inversion of structural boundary conditions while explicitly accounting for uncertainty associated with fracture persistence and rock mass connectivity at depth.

We applied our inversion approach at two sites characterized by toppling rock slabs sharing similar failure mechanisms but in different geological settings. The first site is the Courthouse Mesa slope instability (Utah, USA), which was previously analyzed by Bessette-Kirton et al.^1^. We used their experimental dataset to test our method and produce a direct comparison between deterministic and data-driven inversion strategies. The second site, located in the northwestern region of Malta, features a smaller coastal rock slab isolated by an open rear fracture. Despite its smaller scale, this slope instability exhibits a toppling failure mechanism similar to Courthouse Mesa. Both sites offer excellent accessibility, well-constrained geometries, and clear surficial boundary conditions, representing ideal locations for testing the robustness of our approach. Results indicate that experimentally derived modal parameters can effectively constrain the geometry and mechanical properties of fracture-controlled instabilities, even in the absence of initial geometrical constraints. The identified best-performing models accurately reproduced observed resonance frequencies and three-dimensional mode shapes, revealing fracture-controlled modal deflection patterns and subsurface geometries consistent with field observations. Our findings highlight the potential of operational and numerical modal analysis-based inversion for improved reconstruction of structural models in fracture-controlled rock slope instabilities, without relying on deterministic assumptions. More broadly, our approach can help create detailed geostructural models to be implemented in numerical slope stability analyses, and serve as a foundation for future applications aimed at tracking and relating changes in modal parameters to progressive fracture growth controlling the evolution of unstable rock slopes.

## Study sites and data collection

### Courthouse Mesa

**Fig. 1:**
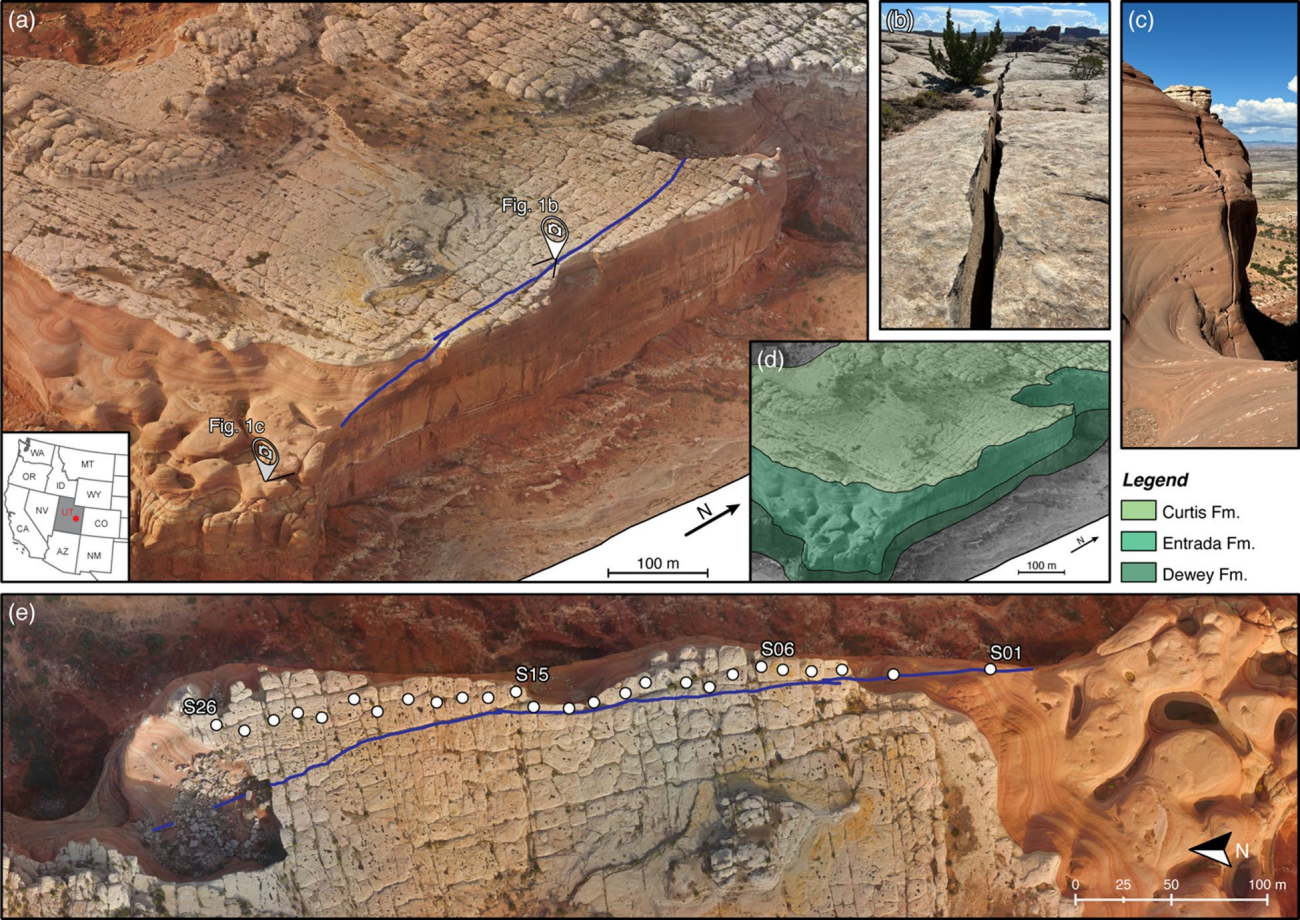
**(a)** Aerial view of Courthouse Mesa (Moab, UT, USA) showing the unstable slab isolated by a ~ 500 m fracture. **b**,** c)** Views of the rear fracture separating the slab and characterized by apertures in the range 0.05–0.5 m. **d)** Geological sketch of the area describing the local setting where the Curtis, Entrada, and Dewey Bridge formations form the vertical wall of the mesa. **e)** Orthophoto of the Courthouse Mesa instability, with locations of the 26 array stations (S01–S26) deployed by Bessette-Kirton et al.^1^. Panels **(a)**, **(d)**, and **(e)** were generated from drone-based photogrammetric imagery processed using Agisoft Metashape (v2.2.3, https://www.agisoftmetashape.com) and subsequently graphically refined and annotated using Adobe Illustrator (v30.1, https://www.adobe.com).

The Courthouse Mesa instability is located 25 km north of Moab, Utah, USA, along the eastern rim of a 100 m high sandstone mesa, where a 500 m long, north-south trending fracture delineates a large rock slab detaching from the plateau (Fig. 1a). The instability consists of a continuous slab bounded by a persistent rear fracture that undergoes east-west oriented opening^1^. The site stratigraphy is well detailed on exposed cliffs, showing the Curtis Formation on top of the mesa, underlain by the Slick Rock Member of the Entrada Sandstone and the Dewey Bridge Member of the Carmel Formation^36^. The competent Entrada Sandstone forms the main body of the slab (~ 70 m thick), while the fine-grained and distorted Dewey Bridge Member provides a mechanically weak and erodible basal layer (Fig. 1d). This contrast in material properties is responsible for cliff undermining, reduced toe support, and progressive development of the instability.

The main fracture at Courthouse Mesa features surficial apertures up to 0.5 m in the southernmost portion of the slab, reaching minimum values of ~ 0.05 m at the northern end (Fig. 1b, c). Local branching and step-overs are observed where the discontinuity intersects thin sandstone layers of the upper caprock. In situ depth measurements using a weighted tape indicated that the fracture extends at least 40 m below the surface in the southern sector, providing a minimum estimate of the fracture depth and delineating a potential failure volume of approximately 0.4 × 10^6^ m^3^. Depth measurements were increasingly uncertain in the northern half of the crack where apertures narrowed requiring a smaller weight that was frequently stuck and had difficulty reaching through branching caprock fractures.

To investigate the dynamic behavior of this failing rock slab, Bessette-Kirton et al.^1^ deployed 26 three-component nodal geophones (5 Hz Fairfield Z-Land) in a dense, linear array with 15 m station spacing covering the length of the exposed slab (Fig. 1e). All sensors were installed at the midpoint between the cliff edge and the fracture, levelled, aligned to magnetic north, and adhered to the bedrock using mounting putty to ensure good ground coupling. Ambient vibrations were continuously recorded at a sampling frequency of 250 Hz during a 17-hour overnight experiment from 16 January 2020, 23:00 UTC to 17 January 2020, 17:00 UTC. However, the longest undisturbed and continuous time block consisted of only 4 h of recordings (17 January 2020, 04:00–08:00 UTC).

## Paradise Bay

**Fig. 2:**
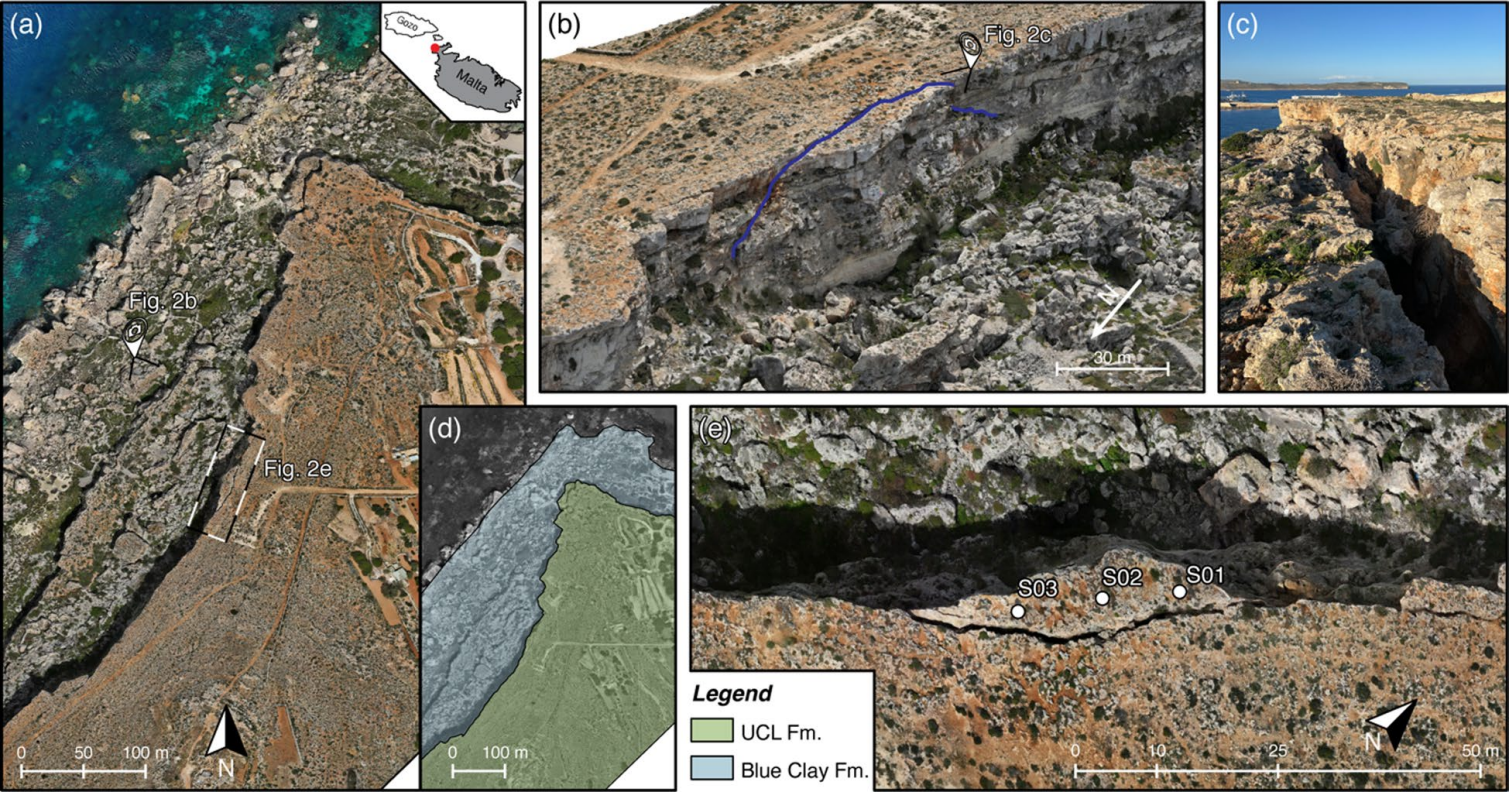
**(a)** Aerial view of the Paradise Bay promontory (Malta) with location of the unstable slab. **(b)** 3D model of the area showing the slab and the open rear fracture (blue line). **(c)** Closer view of the fracture reveals surface apertures of 0.3–0.7 m. **(d)** Geological sketch of the area showing the Upper Coralline Limestone (UCL) Formation resting above the Blue Clay Formation. **(e)** View of seismic array stations S01-S03 aligned with the fracture to capture the dynamic response of the toppling slab. Panels **(a)**, **(b)**, and **(e)** were generated from drone-based photogrammetric imagery processed using Agisoft Metashape (v2.2.3, https://www.agisoftmetashape.com) and subsequently graphically refined and annotated using Adobe Illustrator (v30.1, https://www.adobe.com). The map in panel **(d)** was created using QGIS (v3.16.13, https://qgis.org).

The Paradise Bay promontory is located in northwestern Malta, along the western margin of the Marfa Ridge plateau (Fig. 2a). This area features widespread gravity-induced deformations affecting the cliff-forming limestone plateaus north of Il-Majjistral coast^37,38^. These slope instabilities result from the superposition of a rigid calcarenitic cap rock on a ductile substrate, which predisposes the plateau margins to lateral spreading and, eventually, slope failures^39,40^. The site stratigraphy consists of the Upper Coralline Limestone (UCL), which rests upon the Blue Clay (BC) Formation. The UCL forms a thick, massively bedded calcarenite characterized by sub-horizontal bedding and pervasive fracturing, whereas the BC is composed of highly plastic clays with pronounced ductile behavior, especially when saturated^41–43^.

Along the southern and western rims of Marfa Ridge, high-angle tensile fractures and trenches dissect the UCL plateau, isolating several rock slabs. The aperture of these fractures locally exceeds several tens of centimetres and tends to increase toward the cliff edges, driven by ongoing lateral spreading process^38^. Where steep, vertical fractures trend parallel to cliff edges, rock columns or slabs undergo flexural toppling, while larger compartments may slide or tilt as rigid rock slabs. Similar slope instabilities have been observed at nearby promontories within the same lithological setting across UCL plateaus^44,45^.

We selected an unstable slab located southwest of the Paradise Bay pocket beach (Fig. 2a, b). This slab is entirely composed of the UCL Formation, with a large landslide deposit obscuring the contact with the underlying clays (Fig. 2d). The slab is bounded to the east by a vertical, open fracture approximately 40 m long and characterized by surface apertures in the range 0.3–0.7 m (Fig. 2c). The fracture is continuous and can be traced on the cliff wall northward and southward beyond the slab margins, outlining a slightly trapezoidal geometry when viewed from a distance (Fig. 2b).

We performed point depth measurements using a weighted tape to estimate the persistence of the rear bounding fracture. Results indicate depths up to 14 m in the central area, decreasing toward both lateral ends of the slab. However, these measurements provide only a minimum estimate of fracture depth. The internal fracture surfaces display large-scale undulations that locally prevented the tape from reaching the tip, and several sectors are partially filled with blocks and debris. Moreover, even when we observed no infillings, the narrowing of the fracture at depth may have hindered full penetration of the measuring tape. Consequently, the actual fracture plane likely extends deeper than observed and with a geometry that field observations cannot directly resolve.

To investigate the dynamic response of the slab, we deployed a linear array of three three-component seismometers (2 Hz SARA SL06) aligned to the rear fracture orientation (Fig. 2e). The sensors were spaced 9 m apart to cover the accessible upper surface of the slab, levelled, aligned to magnetic north, and adhered to bedrock using mounting putty to ensure good ground coupling. Ambient vibrations were continuously recorded from 10:00 to 13:30 UTC on 5 February 2025, at a sampling rate of 250 Hz and using synchronized GPS timing. The acquisition was conducted under stable meteorological conditions (i.e., calm winds and no rainfall) to minimize environmental disturbance.

## Methods

### Operation modal analysis

Operational Modal Analysis (OMA) represents a non-invasive method for deriving the modal properties—natural frequencies, damping ratios, and mode shapes— of structures from their response to broadband ambient excitation^46^. OMA methods rely on two key assumptions: (1) the investigated structure behaves as a linear, time-invariant system, and (2) ambient vibrations have white noise characteristics across a broad frequency range. Under these conditions, the dynamic behavior of a structure can be assessed without the need for artificial excitation^21,47^.

Among OMA approaches, Frequency Domain Decomposition (FDD) is a frequency-domain technique that analyzes the spectral content of the system response for identifying its modal properties^48^. The approach uses singular value decomposition (SVD) of the cross-power spectral density (CPSD) matrix, computed from array-based ambient vibration recordings, to decompose the response of a structure into a set of single-degree-of-freedom modal contributions. Compared to traditional peak-picking or polarization analysis techniques, FDD has the advantage of retaining both amplitude and phase information across recordings, enabling reconstruction of modal deformation patterns and discrimination of low-amplitude, closely spaced, or split modes^49,50^. Originally developed for civil and mechanical structures, OMA—and FDD in particular—has been successfully applied in engineering-geological studies, providing insights into the dynamics of natural landforms such as unstable rock slopes and natural arches^1,30,33,51^.

Following the workflow of Häusler et al.^51^, we used FDD to analyze the ambient vibration datasets recorded at both study sites and derive the dynamic properties of the toppling slabs. We first inspected the recordings and retained only the longest undisturbed segments, thus excluding transient signals and anthropogenic disturbances. At Courthouse Mesa, after visual inspection of recordings, only the longest continuous and stationary time window (4 h) was retained for analysis. The selected window is sufficient to ensure stable estimation of cross-power spectral densities and consistent identification of resonance frequencies and mode shapes within the frequency range of interest. At Paradise Bay, the entire acquisition (3.5 h) was retained, as recordings were performed under stable meteorological conditions and showed no evidence of transient disturbances. For this site, the limited number of available sensors may impact the spatial resolution of reconstructed mode shapes and increase uncertainty for higher-order modes, but remains sufficient to resolve the dominant resonance modes targeted in this study. Each recording was then demeaned, detrended, and the instrument response removed. To minimize off-axis motion and enhance sensitivity to resonance modes, we rotated the horizontal components of every array station to radial and transverse directions, aligning these to the main fracture orientation. The CPSD matrix between all recordings was computed at each discrete frequency using Welch’s periodogram^52^, with traces windowed into 100 s, 50% overlapping and 5% cosine-tapered segments. Singular value decomposition was then applied to each matrix to retrieve the corresponding singular values and singular vectors. Peaks in the first singular value spectrum correspond to resonance frequencies, while the corresponding singular vectors describe the associated mode shape^46,48^. To minimize the influence of narrowband or uncorrelated noise, we suppressed persistent monochromatic components following the filtering strategy of Häusler et al.^51^. For each resonance mode, we normalized the complex mode shapes to a reference station and reconstructed the 3D modal vectors through vector composition. Modal vectors, which describe the direction and amplitude of motion at each station, were plotted onto 3D surface models of both rock slabs for visualization and used to compare field-based and numerical modal analysis results.

## Generation of fracture scenarios and numerical modal analysis

To ascertain the mechanical boundary conditions of the selected rock slabs and enhance the interpretability of ambient vibration data, we developed a new workflow integrating stochastic fracture scenario generation with numerical and operational modal analyses. This approach aims to identify sets of boundary condition configurations that best replicate the observed dynamic behavior while accounting for uncertainty in subsurface fracture geometry. Our workflow can generate and test tens of thousands of unique fracture scenarios, each representing admissible boundary condition possibilities.

We used 3D models to reconstruct the geometry of each slope instability. At Paradise Bay, we acquired a set of drone-based, georeferenced images and processed these using photogrammetry to generate a 3D mesh of the area. We manually refined the model by smoothing surface irregularities and trimming the mesh to isolate the slab, considering that the fracture trace marks its rear boundary. The model captures the entire thickness of the Upper Coralline Limestone caprock with its base marking the contact with the Blue Clay Formation. For Courthouse Mesa, we used the 3D model generated from drone-based photogrammetry by Bessette-Kirton et al.^1^. This model also defines the main fracture as the rear boundary of the slab, spanning the Curtis Formation caprock and the Entrada Sandstone, with its base located at the contact with the underlying Dewey Bridge Formation (Fig. 1). Both models thus provide realistic geometric and stratigraphic representations of the unstable rock volumes and the fractures controlling their failure.

**Fig. 3:**
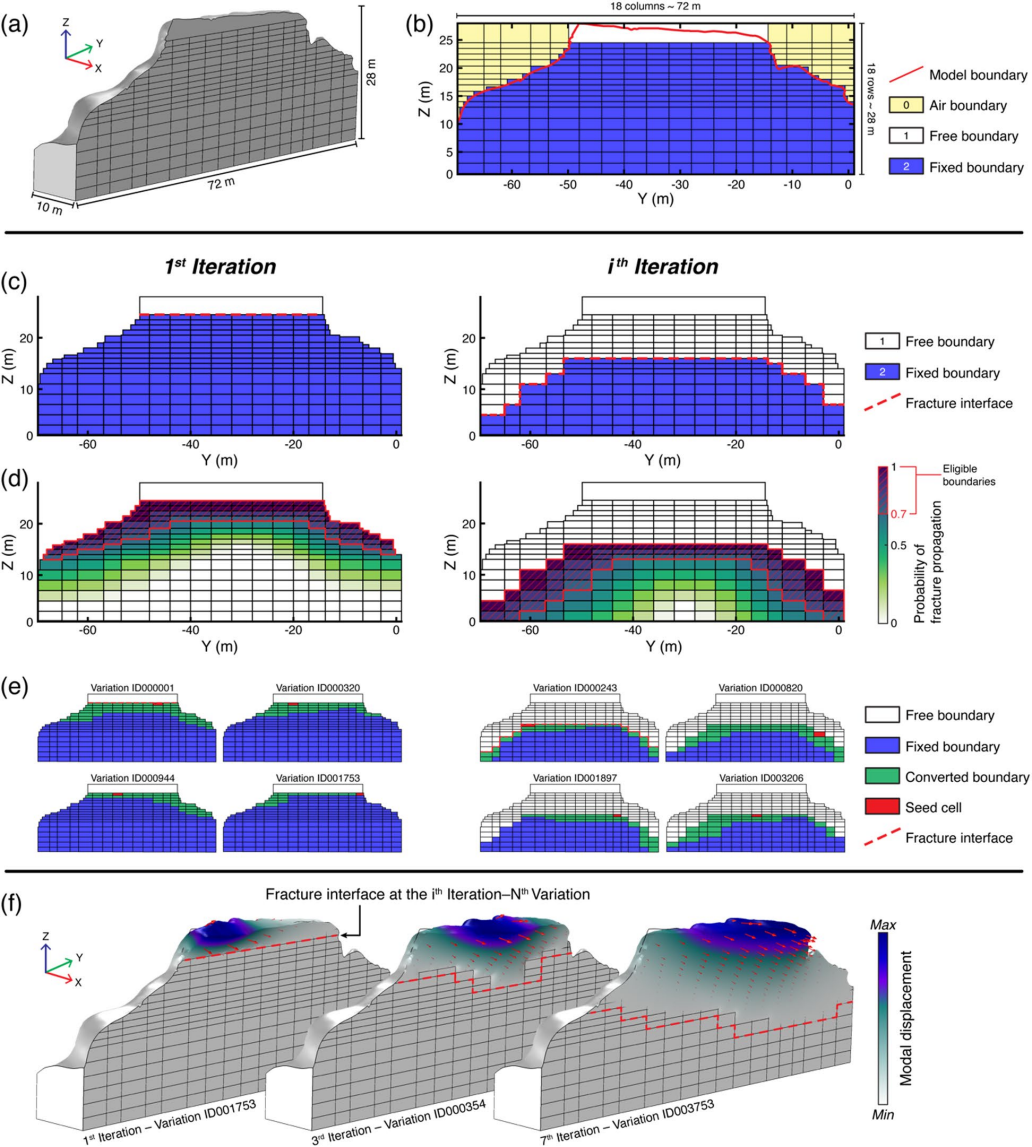
Workflow for the iterative generation and numerical modeling of fracture scenarios. **(a)** Initial model geometry and rear boundary partitioning. **(b)** 2D state matrix encoding the mechanical condition of each boundary element (0: air interface, 1: free boundary, 2: fixed boundary). **(c)** Example of the iterative fracture propagation process simulating the progressive deepening of the fracture interface. **(d)** Probability field applied to the active domain, with higher propagation probability near the current fracture interface (red box). A linearly decaying function is applied to the active domain with values decreasing from 1 at the fracture interface to 0 at the model base; boundaries exceeding the probability threshold (*P* ≥ 0.7) are identified as eligible for fracture propagation. **(e)** Examples of stochastic variations generated at a given iteration, obtained by converting eligible boundary elements (*P* ≥ 0.7) from fixed to free conditions following an eight-connectivity rule. **(f)** Results from numerical eigenfrequency analysis showing the theoretical spatial correspondence between unique fracture scenarios and modal displacement fields.

We then imported each 3D mesh into comsol Multiphysics, where we partitioned the rear boundaries into rectangular grids of 266 and 211 segments for Paradise Bay and Courthouse Mesa, respectively (Fig. 3a). This step allowed us to discretize model boundaries into two-dimensional matrices encoding simplified physical-mechanical states: air interface, intact rock mass (i.e., fixed boundary), and open fracture zones (i.e., free boundary). The adopted binary representation of boundary elements constitutes a mechanical idealization. In this framework, fixed and free boundaries represent end-members of structural coupling rather than exact mechanical states of fracture closure or aperture. Boundaries classified as free correspond to fracture segments with sufficiently low effective stiffness to behave as structurally decoupled, whereas fixed boundaries represent segments coupled to the surrounding rock mass. Partially closed or compliant fractures are therefore implicitly represented when their dynamic response approaches one of these end-member behaviors. After creating the state matrix, we assumed an initial closed-fracture configuration by assigning all boundaries as fixed to explore a wide range of fracture scenarios and avoid potential bias from field observations (Fig. 3b). In line with the kinematics of rigid-block and flexural toppling^53,54^, we assumed fracture initiation with a progressive top-to-bottom propagation. This directional constraint simulates the gravitationally-driven and progressive nature of tensile cracking observed in toppling rock blocks and slabs^55^.

Fracture scenarios were then generated using a stochastic, rule-constrained approach that aimed at introducing controlled variability while maintaining geological and mechanical plausibility. The scenario generation followed an iterative workflow: at each iteration, the fracture interface is deepened by one boundary layer (i.e., one row of the matrix), and multiple randomized scenarios are generated (Fig. 3c). This process continues until the fracture reaches the model base. At each iteration, a linearly decaying probability function is applied to the active domain (i.e., all boundaries located beneath the current fracture interface), with values ranging from 1 at the fracture interface to 0 at the model base. This formulation allows fracture propagation to occur preferentially near the existing free boundaries, with a progressively lower probability of deeper fracture growth. We set and applied a threshold of 0.7 to the probability distributions to cluster all boundaries eligible for transition from fixed to free states, thereby delineating regions where fractures could propagate (Fig. 3d).

Among all eligible boundaries, one boundary was then randomly selected along the fracture interface to initiate fracture growth (i.e., a seed cell), and random, adjacent fixed boundaries that exceed the probability threshold are iteratively freed, following an eight-connectivity rule to ensure geometric continuity within the growing cluster. We also set the proportion of eligible boundaries allowed to vary at each iteration to 5–95% in order to maintain realistic growth while preserving variability across scenarios (Fig. 3e). Each generated configuration is then automatically verified and validated, excluding all variations with isolated or floating free boundaries (i.e., free boundaries surrounded by fixed ones) as well as those failing to meet the prescribed percentage range of fracture growth. Once all valid configurations are obtained for a given fracture interface depth, the iteration continues shifting the interface downward by one additional boundary layer until the base of the model is reached. The resulting set of fracture scenarios encompasses physically plausible geometries, ranging from nearly intact to pervasively fractured conditions, which describe potential boundary conditions for toppling rock slabs.

Each unique scenario was next exported as a state matrix, numerically encoding the mechanical condition of every model boundary, and used to simulate the associated dynamic behavior of the slab. For each model setup, we performed a linear eigenfrequency modal analysis (i.e., in the frequency domain with no input excitation), assuming continuous, homogeneous, and isotropic material properties for both sites. This assumption may not fully capture the in situ heterogeneity or anisotropy of rock masses, but it isolates the effects of boundary conditions on the resulting modal response. For Paradise Bay, we adopted a Young’s modulus of 7.2 GPa, bulk density of 2589 kg m⁻³, and Poisson’s ratio of 0.15, representative of the Upper Coralline Limestone^41^. For Courthouse Mesa, material properties were set to a Young’s modulus of 5 GPa, bulk density of 2200 kg m⁻³, and Poisson’s ratio of 0.20, following the values adopted by Bessette-Kirton et al.^1^ for the site. To compare against field results, we examined the frequency and mode shapes of the measured and modelled resonance modes. In particular, we extracted relative modal displacements in the transverse, radial, and vertical directions at model locations representative of seismic array stations.

To include modal frequencies and mode shapes in the analysis and evaluate the performance of all models in replicating observed dynamic behavior, we designed a combined scalar index that integrates two metrics: (1) the modal assurance criterion (MAC) and (2) the modal frequency ratio (MFR). Together, these parameters help assess agreement between field and numerical results by accounting for both the coherence of modal deformation patterns and relative frequency spacing.

The MAC is a well-established and widely used measure of mode shape collinearity in structural dynamics^56,57^. For a given resonance mode, the MAC between the numerical ($\:{\mathbf{u}}_{\mathbf{i}}$) and field mode shape ($\:{\mathbf{v}}_{\mathbf{i}}$) is computed as:

1 $$\:{\mathrm{M}\mathrm{A}\mathrm{C}}_{\mathrm{i}}=\frac{{\left|{\mathbf{u}}_{\mathbf{i}}^{\mathbf{T}}\bullet\:{\mathbf{v}}_{\mathbf{i}}\right|}^{2}}{\left({\mathbf{u}}_{\mathbf{i}}^{\mathbf{T}}{\bullet\:\mathbf{u}}_{\mathbf{i}}\right)\left({\mathbf{v}}_{\mathbf{i}}^{\mathbf{T}}\bullet\:{\mathbf{v}}_{\mathbf{i}}\right)}$$

This ratio is invariant of the vector magnitude and ranges from 0 (i.e., orthogonal or completely uncorrelated) to 1 (i.e., identical mode shapes). In practice, MAC evaluates how well the mode shapes align in direction, regardless of their relative amplitude. This property is essential when comparing numerical and field mode shapes that may differ in scale due to normalization or measurement units, as it ensures a consistent and physically meaningful assessment of their directional similarity. In this study, we computed the MAC for every model and resonance mode with respect to the corresponding field-derived mode shape. For each model, the resulting MAC values were then averaged across all resonance modes to obtain a scalar index representing the overall model’s ability to replicate the observed mode shapes.

However, MAC only captures the similarity in spatial deformation patterns and is insensitive to modal frequency. Therefore, to assess frequency agreement, while mitigating the influence of absolute frequency shifts caused by uncertain material properties, we computed modal frequency ratios (MFRs) relative to the fundamental frequency:

2 $$\:{\mathrm{MFR}}_{\mathrm{i}}=\frac{{f}_{i}}{{f}_{1}}\:,$$

where $\:{f}_{1}$ is the fundamental resonance frequency and $\:{f}_{i}$ is the frequency of the *i*-th resonance mode. This normalization removes the influence of global stiffness scaling, enabling comparison of modal frequency spacing, which is primarily governed by geometry and boundary conditions rather than absolute material properties^58^. For each model and field dataset, the MFRs are assembled into a vector $\:\mathrm{r}$:

3 $$\:\mathbf{r}=\left[{MFR}_{2},{MFR}_{3},...,{MFR}_{N}\right],$$

where N is the number of matched resonance modes considered in the analysis. We quantified the mismatch between modelled and measured MFRs by computing the Euclidean distance at every resonance mode (E_MFR_):

4 $$\:{E}_{MFR}^{m}=\:{\parallel{{{{\mathbf{r}}}}}^{m}-{\mathbf{r}}^{f}\parallel}_{2}=\sqrt{\sum\:_{i\:=\:2}^{N}{\left({MFR}_{i}^{m}-{MFR}_{i}^{f}\right)}^{2}}$$

where superscripts ^(m)^ and ^(f)^ denote the numerical model and field data, respectively. The MFR mismatch was then min–max normalized across the full ensemble of tested numerical models for each site, yielding a scalar index ranging from 0 (i.e., perfect match) to 1 (i.e., highest mismatch within the dataset):

5 $$\:{\epsilon\:}_{MFR}^{m}=\:\frac{{E}_{MFR}^{m}-\mathrm{m}\mathrm{i}\mathrm{n}\left({E}_{MFR}\right)}{\mathrm{m}\mathrm{a}\mathrm{x}\left({E}_{MFR}\right)-\mathrm{m}\mathrm{i}\mathrm{n}\left({E}_{MFR}\right)}$$

Since the MAC and MFR capture distinct aspects of modal behavior similarity, they are complementary rather than independent. To synthesize these parameters into a single, balanced metric of overall model performance, we defined a Weighted Combined Similarity (WCS) index as:

6 $$\:\mathrm{W}\mathrm{C}\mathrm{S}\:=\:{\mathrm{w}}_{\mathrm{M}\mathrm{A}\mathrm{C}}\overline{\mathrm{M}\mathrm{A}\mathrm{C}}\:+{\mathrm{w}}_{\mathrm{M}\mathrm{F}\mathrm{R}}\left(1-{\epsilon\:}_{MFR}\right)$$

where $\:{\mathrm{w}}_{\mathrm{M}\mathrm{A}\mathrm{C}}$ and $\:{\mathrm{w}}_{\mathrm{M}\mathrm{F}\mathrm{R}}\:$are the weights reflecting the relative confidence placed in mode shape and frequency scaling, and $\:\overline{\mathrm{M}\mathrm{A}\mathrm{C}}$ is the arithmetic mean of MAC values across all resonance modes of every model. We adopted a weighting scheme that prioritizes mode shape similarity over relative frequency mismatch ($\:{\mathrm{w}}_{\mathrm{M}\mathrm{A}\mathrm{C}}$ = 1, $\:{\mathrm{w}}_{\mathrm{M}\mathrm{F}\mathrm{R}}$ = 0.3) because mode shapes are more sensitive to changes in boundary conditions, whereas modal frequencies may be influenced by heterogeneous material properties. This strategy reflects a conservative bias toward mode shape, which is generally described as more robust in ambient vibration studies of engineering structures^47^. We finally used the WCS to rank all models based on their performance. In addition to identifying the best-performing model for each site (i.e., the model with the highest WCS score), we evaluated all models that exceeded the 90th and 99th percentiles of the WCS distributions to compare the top-performing model ensembles. The inversion framework does not seek a unique fracture geometry. Instead, it constrains a family of boundary-condition configurations that are dynamically consistent with the observed modal parameters. Because different fracture geometries may reproduce similar resonance frequencies and mode shapes, multiple admissible solutions can achieve comparable similarity scores. For this reason, model performance is evaluated at the ensemble level rather than through a single best-fitting model. Persistent structural features that recur across high-ranking model subsets are interpreted as constraints on fracture persistence, whereas features that vary strongly across models reflect non-uniqueness and residual uncertainty.

This approach enables us to evaluate the consistency of fracture geometries across top-ranked models and to identify recurring patterns relevant for structural characterization of the sites. Finally, we explored the results through targeted validation checks, including comparisons of normalized modal amplitudes and 3D angular deviations between field and numerical modal vectors, as well as coherence between the probabilistic fracture configurations compiled from all high-ranked models and fracture depths measured in the field.

## Results

### Operational modal analysis

**Fig. 4:**
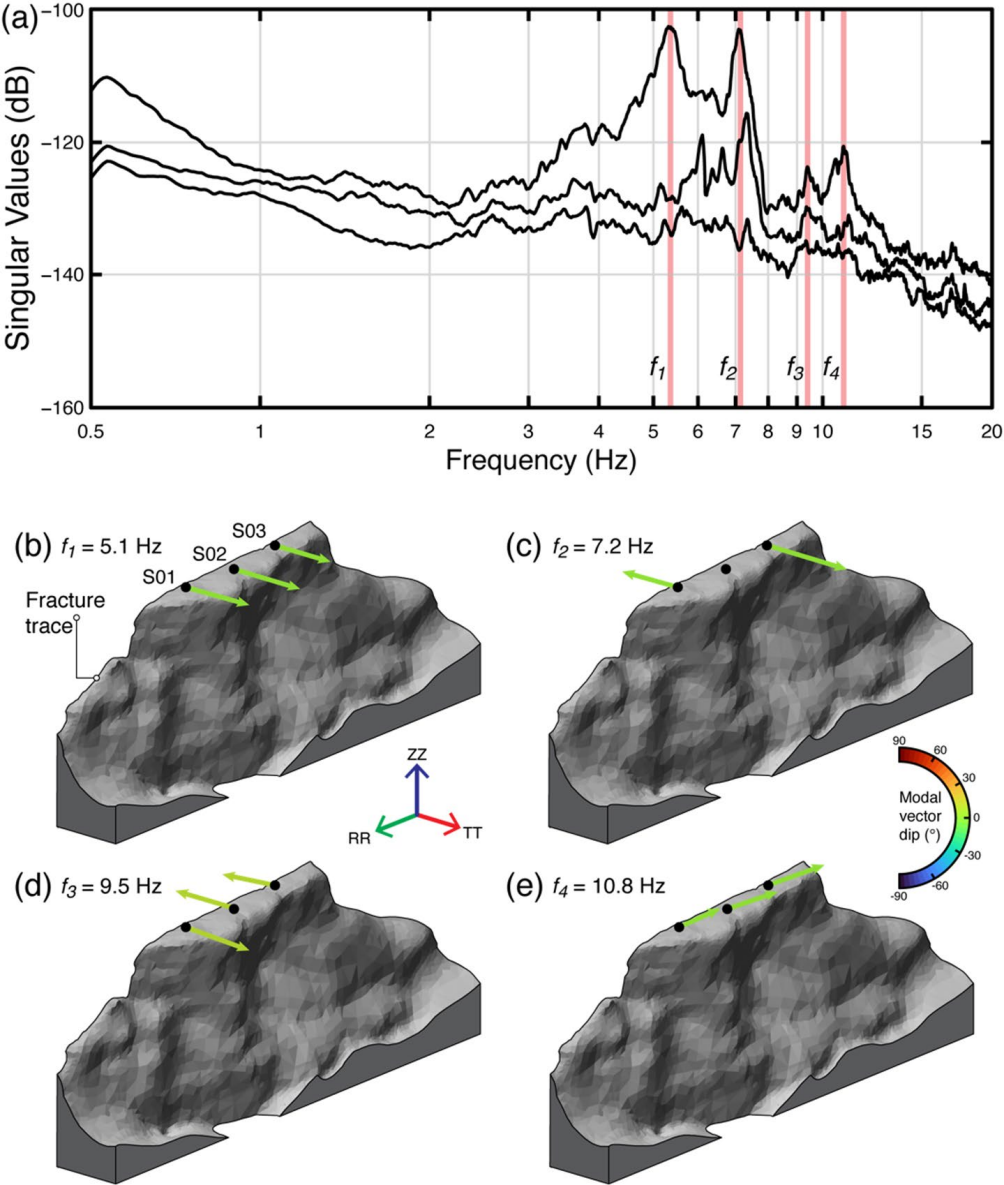
Results of operational modal analysis for the Paradise Bay rock slab. (**a)** Spectrum of the first three singular values, where red shadings indicate the four identified resonance modes. (**b–e)** 3D mode shapes of all resonance modes with color-coded modal vectors according to their dip angle.

**Fig. 5:**
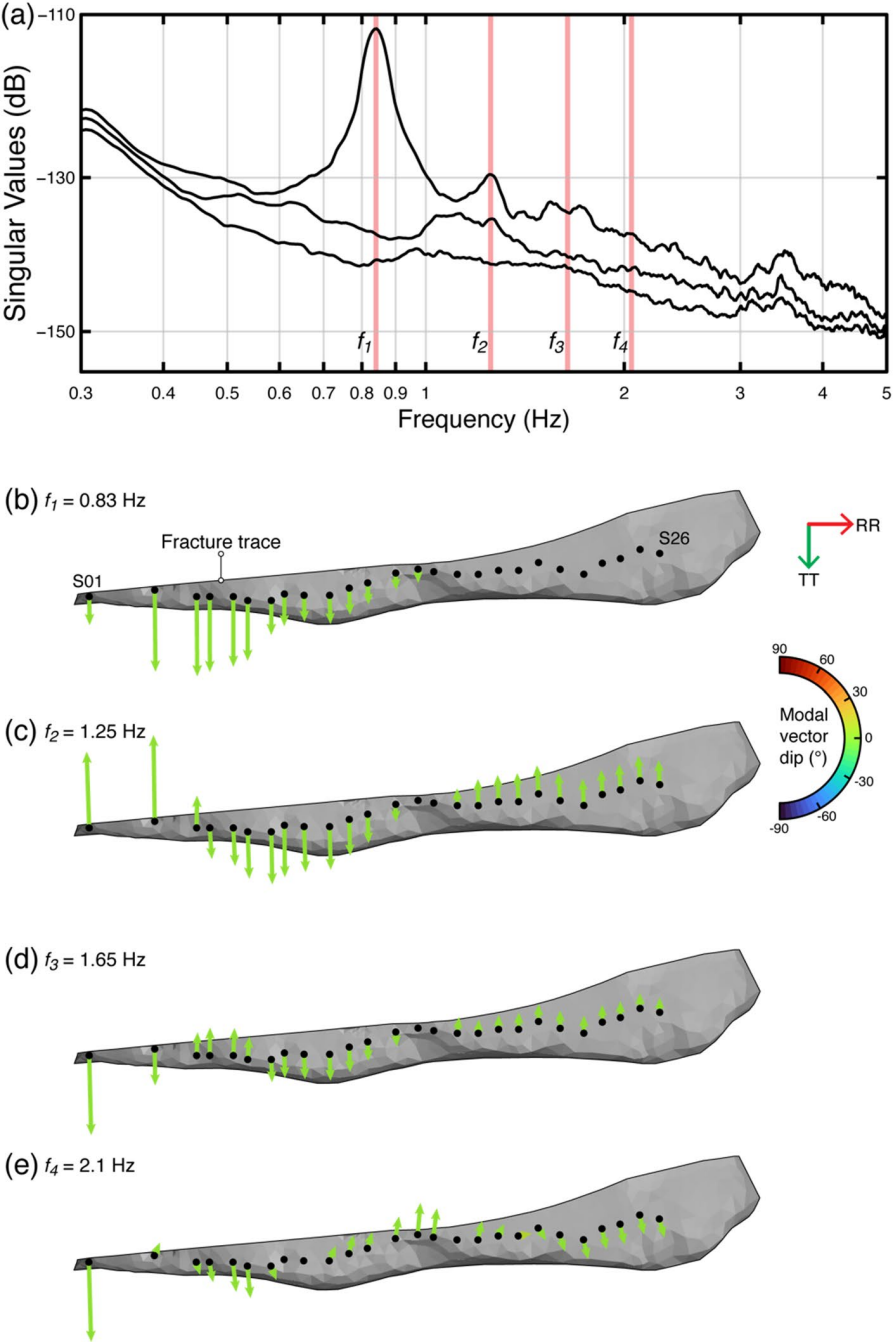
Results of operational modal analysis at Courthouse Mesa. **a)** Spectrum of the first three singular values, where red shadings indicate the four identified resonance modes. **b–d)** 3D mode shapes of all resonance modes with color-coded modal vectors according to their dip angle.

Ambient vibration analysis resolved well-defined resonance modes characterized by coherent modal vectors and consistent polarization patterns at both sites. Peaks in the singular value curves mark the main resonance frequencies, from which the corresponding mode shapes were extracted (Figs. 4a and 5a). We identified four resonance modes at Paradise Bay (Fig. 4). The first mode (*f*_*1*_ = 5.1 Hz) corresponds to a fundamental out-of-plane bending mode, dominated by slab bending from its base (Fig. 4b). The second (*f*_*2*_ = 7.2 Hz) and third (*f*_*3*_ = 9.5 Hz) modes represent higher-order torsional modes with node points between stations S01 and S03 (Fig. 4c, d). The fourth mode (*f*_*4*_ = 10.8 Hz) shows in-plane bending with modal vectors parallel to the rear fracture (Fig. 4e). Overall, the first three modes display sub-horizontal patterns of modal deformation that are perpendicular to the fracture strike, while the fourth mode shows modal vectors aligned with the fracture trace.

At Courthouse Mesa, our analysis confirmed results of Bessette-Kirton et al.^1^. While the rock slab exhibits more than six resonance modes, we elected to focus only on the first four, which are the dominant peaks in the SVP and thus the most structurally relevant (Fig. 5a). All four modes represent out-of-plane bending modes of increasing order (*f*_*1*_ = 0.83 Hz, *f*_*2*_ = 1.25 Hz, *f*_*3*_ = 1.65 Hz, *f*_*4*_ = 2.1 Hz), distinguished by an increasing number of node points (Fig. 5b–e). Modal vectors are dominantly oriented in the transverse direction, or normal to the rear fracture, and show greater relative displacements at the southern end of the slab. The fourth mode features slightly rotated modal vectors with respect to the fracture trace in the central part of the slab, highlighting a greater radial contribution to this mode shape.

### Numerical modal analysis

**Fig. 6:**
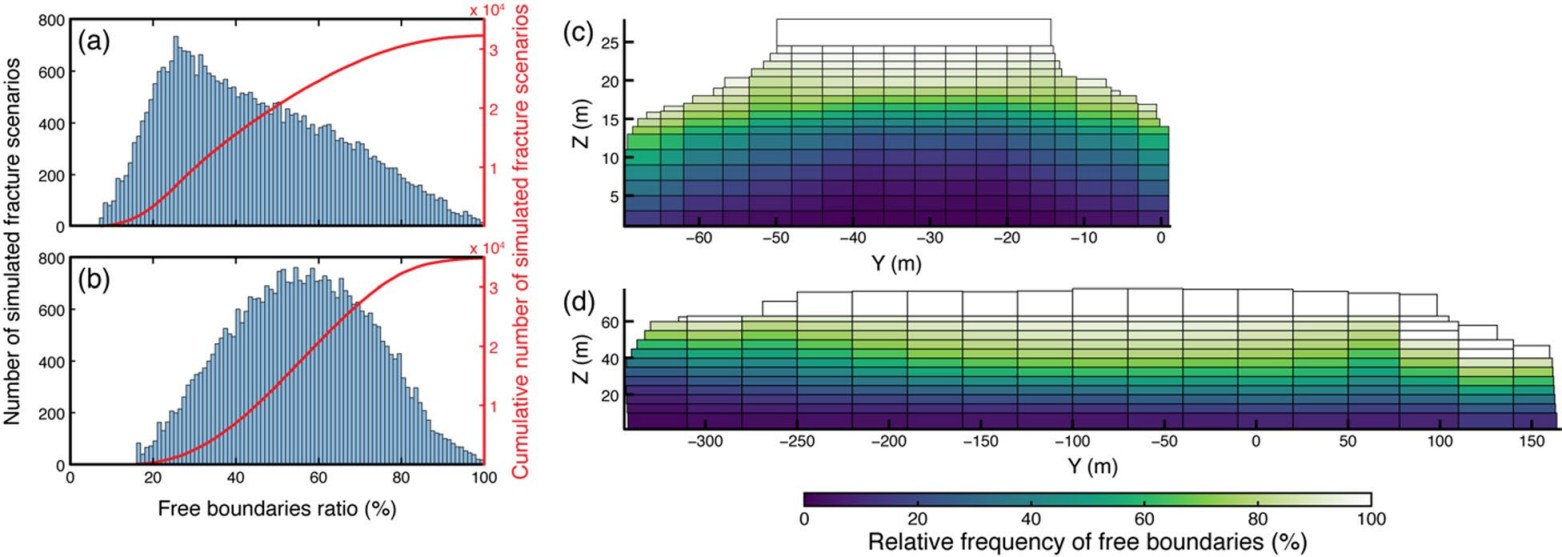
Distribution of generated fracture scenarios for Paradise Bay **(a)** and Courthouse Mesa **(b)** as a function of the free-boundary ratio, defined as the ratio between the cumulative area of free boundaries and the total rear boundary area. Red lines indicate the cumulative number of simulated scenarios. Spatial distribution of the relative frequency of free boundaries for Paradise Bay **(c)** and Courthouse Mesa **(d)**, obtained by compiling all simulated fracture scenarios. Values of 100% indicate that boundaries were retained free across all simulated configurations, whereas 0% corresponds to boundaries that remained fixed.

We generated and tested 32,280 and 34,835 unique fracture scenarios for the Paradise Bay and Courthouse Mesa instabilities, respectively (Fig. 6). These model ensembles contain substantial variability in simulated boundary conditions, with the overall percentage of free boundaries ranging from less than 10 to more than 80% (Fig. 6a, b). For both sites, we obtained a nearly continuous distribution of free-boundary ratios, defined as the ratio between the cumulative area of free boundaries and the total rear-boundary area, with a greater number of intermediate states of limited fracture persistence (Fig. 6c, d). This outcome confirms that the stochastic scenario generation successfully sampled a broad spectrum of boundary conditions, from nearly fully coupled to extensively detached rock slabs. The overall spread of free-boundary ratios is greater for Courthouse Mesa, simulating greater structural variability and a wider range of possible fracture geometries. In contrast, fracture geometries for Paradise Bay cluster within a narrower interval. Collectively, the two model ensembles provide a comprehensive representation of potential rear fracture configurations to be explored and tested.

**Fig. 7:**
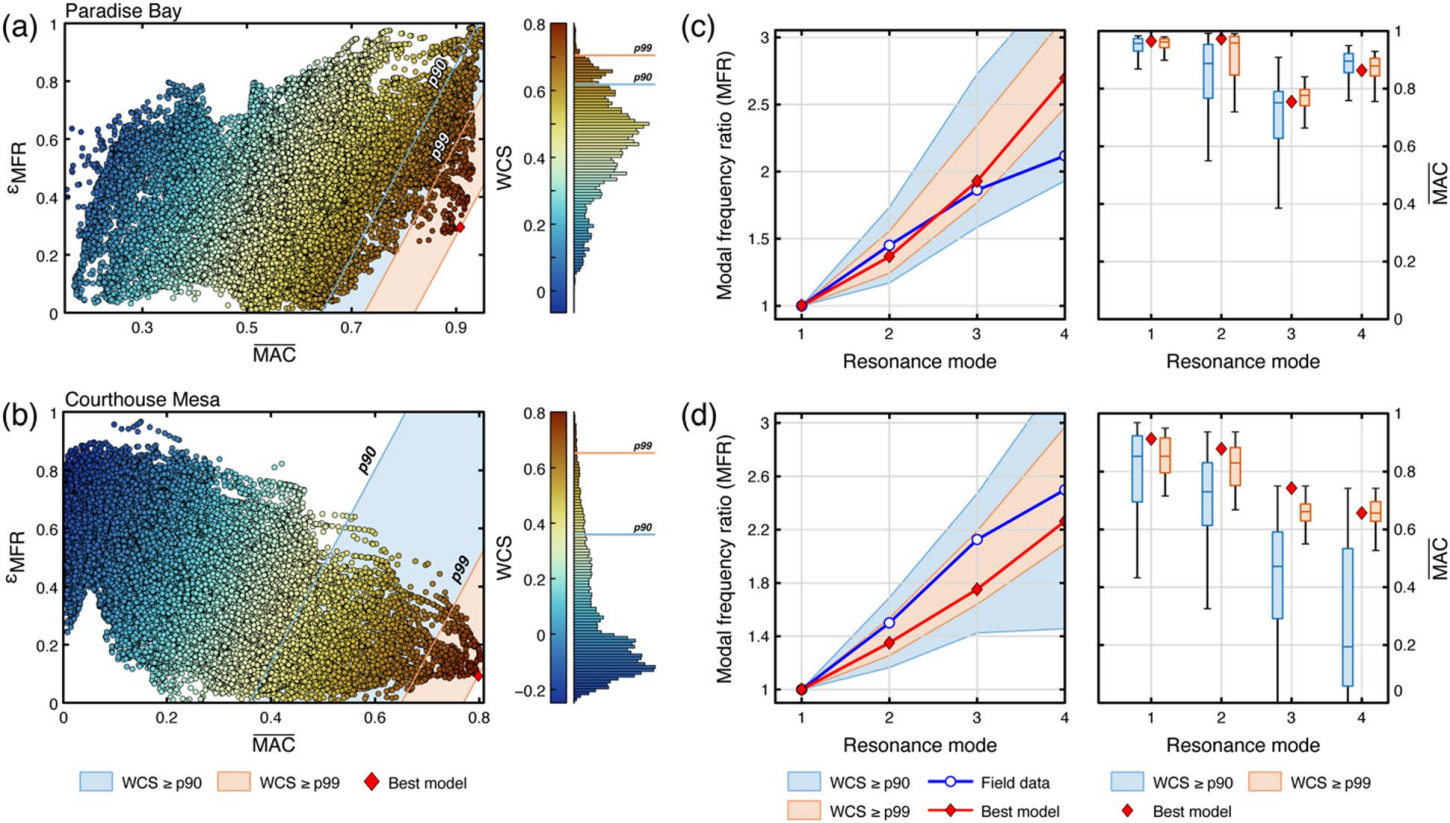
Distribution of all simulated models in the $\overline{\mathrm{M}\mathrm{A}\mathrm{C}}$–$\:{\epsilon\:}_{MFR}$ space for Paradise Bay **(a)** and Courthouse Mesa **(b)**. All points are colored according to their WCS score, with shaded areas highlighting the regions above the 90th and 99th percentiles (p90 and p99). Red diamonds indicate the best-performing model for each site. Numerical models featuring high $\overline{\mathrm{M}\mathrm{A}\mathrm{C}}$ and low $\:{\epsilon\:}_{MFR}$ yield higher WCS values, reflecting greater mode shape and relative frequency spacing similarity. Comparison between field and numerical results for the first four resonance modes **(c**,** d)**. Left panels show the modal frequency ratios (MFRs) for field data (blue line), best-performing (red line), p90 (orange area), and p99 (blue area) model subsets; the shaded areas represent the variability among models within each percentile range. The right panels describe the corresponding distribution of average MAC values for each resonance mode. Together, panels c and d highlight the relative consistency of model–field data agreement across the considered resonance modes.

Comparison between field and numerical modelling results highlights the subset of boundary configurations that best reproduce the field results at both sites. Figure 7 summarizes the performance of all tested models: at Paradise Bay, higher MAC values are associated with larger relative frequency mismatches, whereas at Courthouse Mesa, the opposite trend emerges. (Fig. 7a, b). Models within the 90th and 99th percentiles of the WCS distribution (Fig. 7a, c) define the most consistent boundary configurations (i.e., the top 10 and 1% model ensembles, respectively). The distribution of modal frequency ratios and mode-shape similarities for each resonance mode is summarized in Fig. 7b and d. For both sites, we observe that the top-performing models reproduce field data across successive resonance modes with limited dispersion, confirming their plausibility. Only the 90th percentile groups show larger data dispersion, especially at higher modes. For mode shape similarity, Paradise Bay shows consistent MAC values that remain higher than 0.75 across all simulated modes (Fig. 7b), while Courthouse Mesa shows increasing mismatch at modes three and four (Fig. 7d). This reduction in coherence with mode order is consistent with increasing sensitivity of modal behavior to local, small-scale structural and material heterogeneities that can create complex modal deformation patterns.

**Fig. 8:**
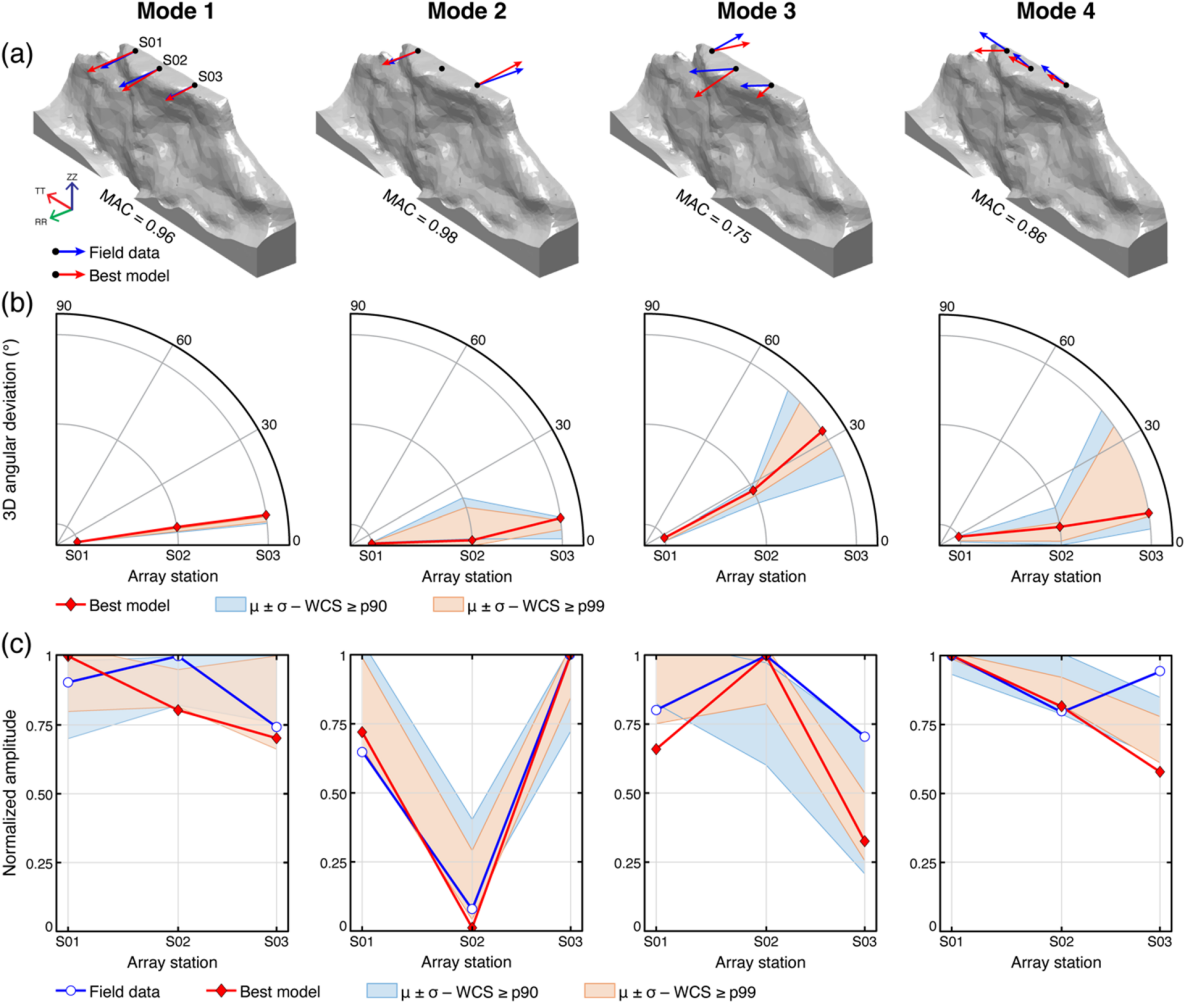
Comparison between field and numerical modal results for the four resonance modes of Paradise Bay. **(a)** 3D modal vectors of the field (blue arrows) and the best-performing model (red arrows). The MAC value for each resonance mode highlights the similarity in mode shapes. **(b)** 3D angular deviation between field and numerical modal vectors at each station, with red lines representing the best-performing model and shaded areas indicating the mean and standard deviation of models within the 90th (blue) and 99th (orange) WCS percentiles. **(c)** Comparison of modal displacement amplitudes from field (blue) and numerical (red) data, normalized by the maximum absolute modal vector amplitude at each mode to enable visual comparison of relative deformation patterns along the array.

**Fig. 9:**
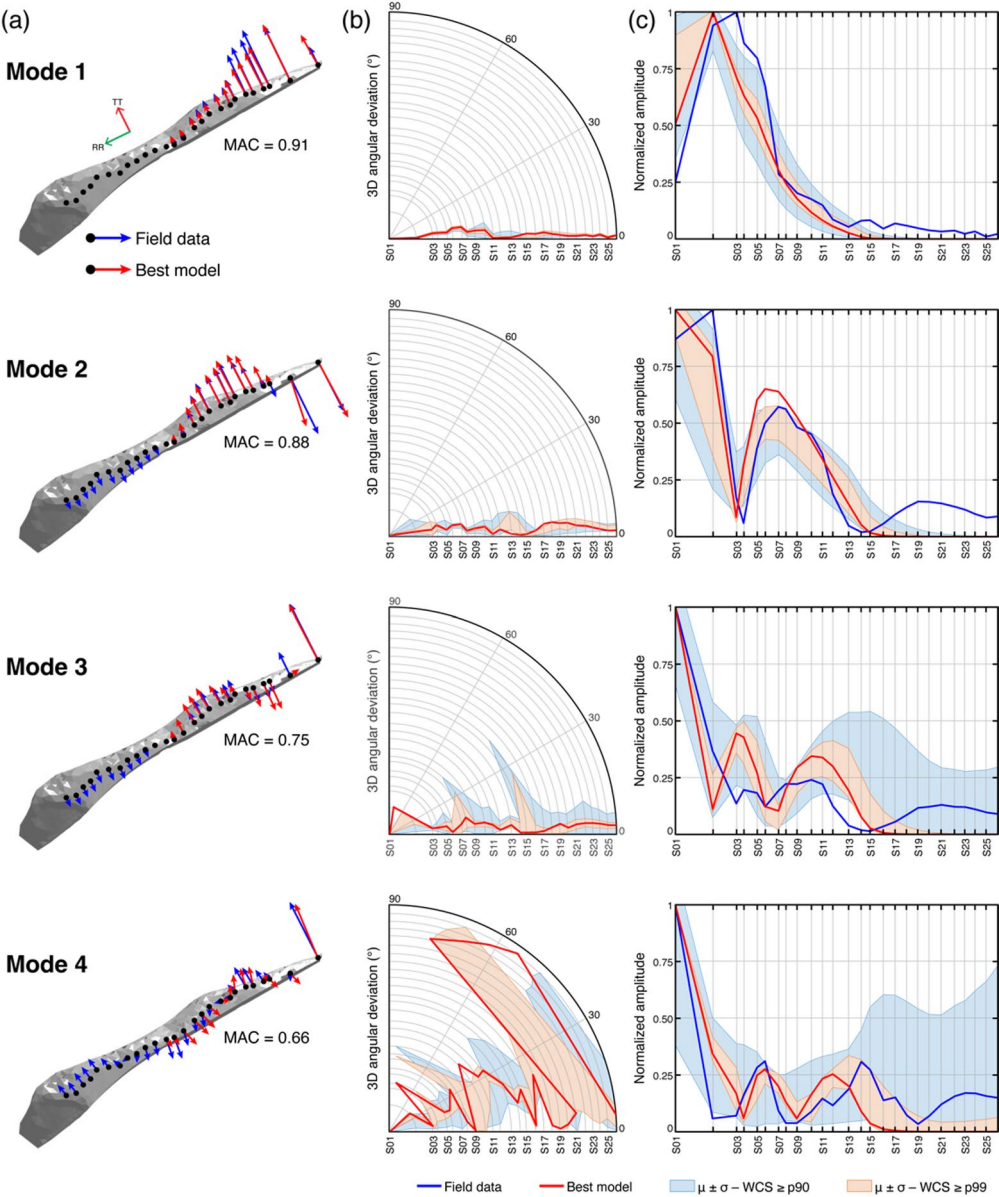
Comparison between field and numerical modal results for the four resonance modes at Courthouse Mesa. **(a)** 3D modal vectors of the field (blue arrows) and best-performing model (red arrows). **(b)** 3D angular deviation between field and numerical modal vectors at each station, with red lines representing the best-performing model and shaded areas indicating the mean and standard deviation of models within the 90th (blue) and 99th (orange) WCS percentiles. **(c)** Comparison of normalized modal displacement amplitudes from field (blue line) and numerical (red line) data, normalized by the maximum absolute modal vector amplitude at each mode to enable visual comparison of relative deformation patterns along the array.

Analysis of 3D angular deviations and normalized amplitudes of field and numerical modal vectors enabled us to deduce strong correspondence in mode shapes across the two study sites and to evaluate the coherence and stability of the top-performing models (Figs. 8 and 9). At Paradise Bay, numerical models accurately reproduced the observed resonance modes. The first two modes exhibit similar orientations of field and numerical modal vectors, with MAC values exceeding 0.9 and angular deviations less than 30°. The 99th percentile subset outlines narrow envelopes around these directions, indicating that top-performing models converge toward a common modal vector direction (Fig. 8b). The normalized amplitude distributions follow a similar pattern, reproducing the relative displacement at each array station and predicting the same locations of node points (Fig. 8c). The third and fourth modes show greater variability, consistent with their more complex deformation patterns, however, the dominant polarization features are resolved.

At Courthouse Mesa, the agreement between numerical and field modal vectors extends through the first three modes (Fig. 9a), with MAC values generally higher than 0.7 (Fig. 7d). Numerical models consistently replicate the orientation and magnitude of experimental modal vectors, with minor angular deviations ranging 0–30° and stable relative amplitudes. Substantial divergence emerges in the fourth mode, however, where broader directional and amplitude differences, with 3D angular deviations exceeding 60° in the northern portion of the slab, indicate limitations in the model’s ability to replicate the observed dynamic behavior (Fig. 9b, c). Across all modes, field measurements consistently indicate higher modal amplitudes in the northern portion of the slab than predicted by numerical models. This discrepancy may arise from external excitation, such as wind, local energy sources, or topographic amplification along the Mesa rim.

**Fig. 10:**
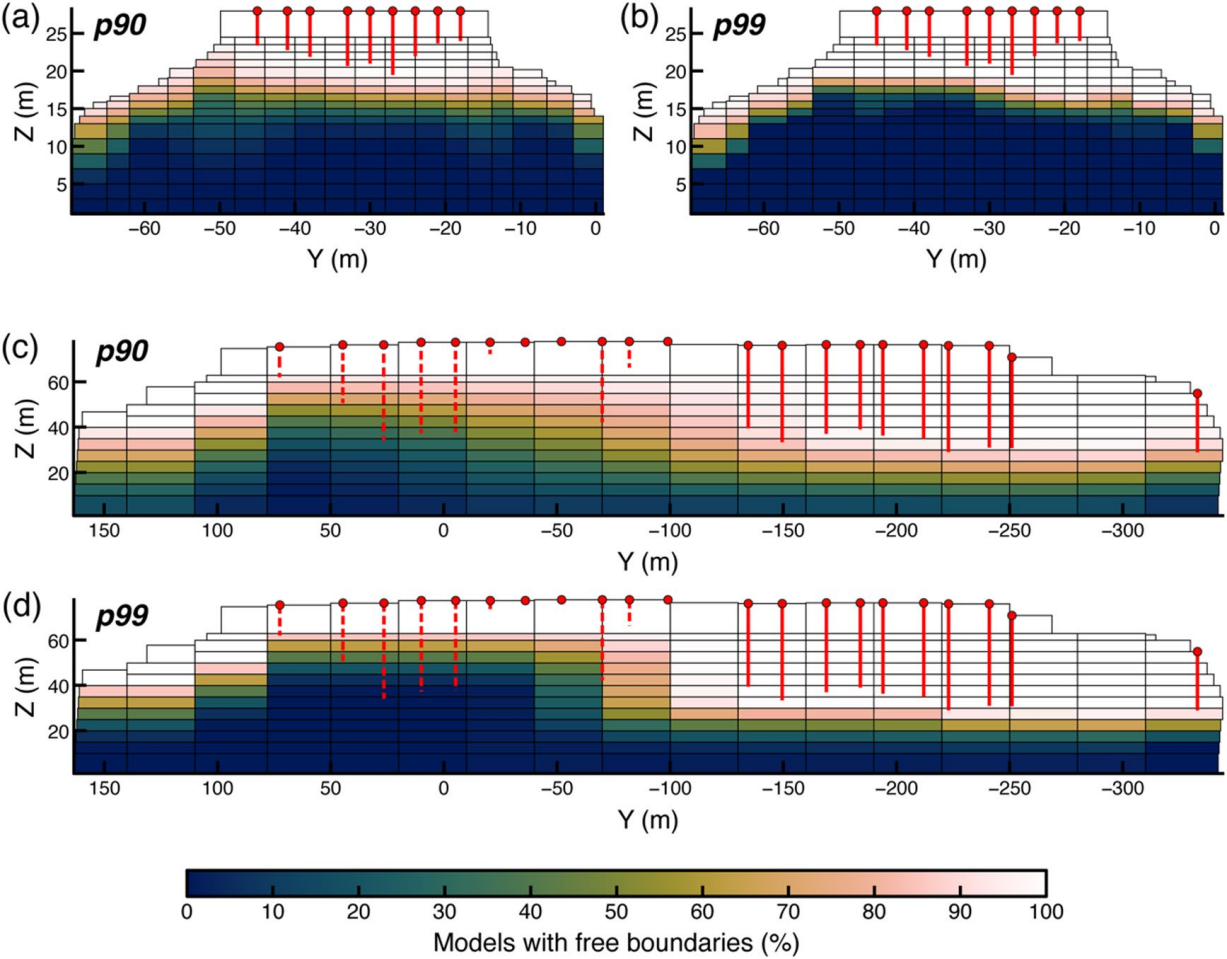
Probabilistic representation of fracture persistence across top-performing models for Paradise Bay **(a**,** b)** and Courthouse Mesa **(c**,** d)**, showing the spatial distribution of boundaries for the 90th and 99th WCS percentiles. The colorscale indicates the relative frequency (percentage) of models within each percentile subset in which each boundary was free, from 0 (always fixed) to 100% (always free), and therefore represents a conditional, ensemble-based measure rather than an absolute fracture probability. Vertical red lines mark the fracture depths measured in the field. For Courthouse Mesa, dashed lines denote fracture depth measurements on the north end of the slab characterized by substantial uncertainty **(c**,** d)**. The maps highlight the most recurrent detachment zones among top-performing model configurations, delineating areas of persistent structural decoupling.

Overall, our results indicate that the top-performing models successfully reproduce the resonance frequencies and mode shapes of the two unstable slabs, providing a reliable basis for assessing their underlying boundary conditions. Therefore, we derived a probabilistic representation of fracture geometries by compiling the relative frequency of free boundaries for the top 10 and 1% of all tested models (90th and 99th WCS percentiles) at each site. At Paradise Bay, both subsets indicate a well-defined zone of persistent detachment along the upper part of the rear boundary, where more than 90% of the top-ranked models retain free-boundary conditions (Fig. 10a, b). This zone aligns with the fracture depth observed in the field, confirming the results from in situ measurements. At Courthouse Mesa, reconstructed boundary conditions are discontinuous along the central and southern sectors of the slab, whereas the northern portion remains largely fixed across the top-performing model ensembles (Fig. 10c, d). We observe a broader distribution of probabilities that reflects a wider range of admissible boundary configurations.

The comparison between field-measured fracture depths and numerical results shows good agreement in the southern and central regions of the slab (i.e., right and central sectors of the model in Fig. 10c and d), supporting the consistency between numerical and observational constraints. In contrast, the northern sector of the slab shows limited agreement between numerical results and field observations, as all models predict shallower fracture interfaces. However, as noted, field measurements of crack depth in this area were challenging and are considered less reliable.

## Discussion

Resonance modes identified at Paradise Bay and Courthouse Mesa show clear evidence of fracture control on the dynamic behavior of these unstable rock slabs. At Paradise Bay, the four identified resonance modes describe a flexural toppling instability characterized by bending and torsional modes about an adhered base, consistent with the local geological setting. The alignment of modal vectors for modes 1–3, normal to the fracture strike, suggests that the observed dynamic behavior is controlled by the structural decoupling of the unstable volume from the plateau, while mode 4 shows deformation vectors parallel to the fracture trace. Similarly, the first four resonance modes observed at Courthouse Mesa represent a sequence of out-of-plane bending modes featuring an increasing number of node points with mode order. The direction of modal vectors is generally perpendicular to the main rear fracture, and the large amplitude of relative displacements in the southern portion of the slab suggests asymmetric crack depth conditions, confirming the structural model of a partially detached slab proposed by Bessette-Kirton et al.^1^.

FDD results from both sites support the use of array-based ambient vibrations as a best practice for resolving the dynamic behavior of rock slopes and natural landforms. Compared with single-station or sparse-array ambient vibration surveys^32,59,60^, dense arrays have the advantage of enabling reconstruction of spatially detailed 3D mode shapes^22,30,33,49^, results that are essential for constraining geostructural models and evaluating the validity of potential boundary configurations. Nonetheless, dense array data cannot provide more than qualitative insights into the deep geometry of fractures controlling rock instabilities. While modal analysis can help investigate the instability extent and volume involved, highlighting situations of partial structural decoupling^16,51,61^, the results do not allow for fracture characterization at depth. For this reason, seismic array datasets provide the experimental foundation on which numerical modelling must build to invert for obscured boundary conditions.

We developed a stochastic, rule-based numerical modelling approach to invert the observed dynamic behavior and reconstruct subsurface fracture geometries of the toppling slabs. By generating more than thirty thousand unique fracture scenarios per site, we ensured that a continuum of boundary conditions—from fully bonded to completely detached—was represented. Among these, only small subsets of top-performing models accurately reproduced the measured modal properties. From reconstruction of probabilistic fracture representations, the clustering of these models toward well-defined configurations suggests that the inversion procedure is stable and selective (Fig. 10). Nonetheless, the inversion remains intrinsically non-unique. Ambient-vibration modal parameters integrate the combined effects of fracture geometry, material properties, and boundary conditions, and therefore cannot uniquely resolve subsurface fracture configurations. For this reason, results are expressed as admissible model ensembles rather than deterministic solutions. To this end, the probabilistic fracture-persistence maps enable the identification of boundary elements that recur consistently across top-performing models, whereas spatially variable patterns reflect residual uncertainty in boundary conditions.

A key element supporting the reliability of this procedure is independent validation provided by field observations. The boundary conditions inferred from numerical modelling are consistent with field depth measurements from both sites, despite those measurements not being used as input for initial model setup. The agreement between reconstructed boundary conditions and surveyed fracture depths not only validates the approach but also demonstrates that ambient vibration data can yield information on subsurface fracture geometries without relying on field-based assumptions of fracture geometry. However, validation of fracture configurations is necessarily qualitative and focuses on geometric consistency rather than quantitative depth matching. The correspondence between zones of high inferred fracture persistence and field-measured fracture depths supports the plausibility of the numerical results, while acknowledging that exact fracture depths cannot be uniquely resolved from the available data. This validation step also defines the main conceptual distinction from the deterministic approach adopted by Bessette-Kirton et al.^1^. In this previous study, the geometry of the simulated fracture scenarios for Courthouse Mesa was prescribed based on field depth measurements, thereby limiting model variability to predefined possibilities. On the contrary, we relied solely on results from ambient vibration array data to infer boundary geometries, using field data for validation and thereby avoiding potential bias arising from the sparse, locally variable nature of fracture depth measurements.

Comparison between numerical and field results highlights both the strengths and limitations of our approach. Overall, the top-performing models for both sites closely reproduced the main observed modal properties (i.e., frequency ratios and mode shapes), suggesting that the initial assumptions of homogeneous material properties and simplified boundary conditions capture the sites’ bulk dynamic behavior. However, deviations in higher-order modes indicate increasing influence of local heterogeneities not implemented in our numerical models. For example, in the case of Paradise Bay, differences observed in mode shapes and frequency ratios for the third and fourth resonance modes (Figs. 7a and b and 8) likely stem from the assumption of a planar fracture trace and homogeneous material properties, which may fail to replicate the local cliff-forming limestones. In real rock masses, heterogeneous material properties can influence modal parameters. With the proposed approach, such uncertainties may therefore lead to alternative subsurface fracture configurations that yield comparable agreement in dynamic behavior. However, the use of a multi-metric evaluation criterion mitigates sensitivity to absolute stiffness contrasts, emphasizing geometric and kinematic constraints imposed by fracture persistence. As a result, the inferred fracture configurations should be interpreted as admissible representations based on simplified material property assumptions, rather than precise reconstructions of fracture geometry. At Courthouse Mesa, the divergence in modal vector orientations and amplitude at the fourth resonance mode (Fig. 9) might reflect topographic amplification or localized resonances along the Mesa rim that are not captured by our numerical models. Higher-order modes can be particularly informative for detecting and monitoring structural change, yet caution must be applied in their interpretation as their low energy and complex mode shapes make them more unstable indicators than corresponding fundamental modes^26,30,62–64^.

From a methodological perspective, one of the most significant advantages of our approach lies in its non-invasive nature and independence from initial deterministic assumptions of fracture geometry or persistence. Our technique integrates ambient-vibration modal analysis—regardless of the methods used to identify resonance modes—with numerical modelling, leveraging automated generation of admissible fracture scenarios and a multi-metric evaluation criterion. In particular, combining different metrics for mode shape and frequency-ratio similarity provides complementary constraints, ensuring that model evaluation reflects accurate geostructural conditions rather than simple frequency matching, which is more suited when boundary conditions are clear^24^. However, the completeness of the simulated fracture scenarios remains paramount. To ensure reliable numerical results, the generated scenarios must span a broad spectrum of boundary conditions, as complete exploration is unfeasible. Even considering 20 boundaries instead of 200 would require more than 10^18^ combinations of free and fixed state assumptions. Such a growth demands targeted reduction strategies, retaining only fracture configurations that satisfy both structural and kinematic compatibility with the considered failure mechanism.

**Fig. 11:**
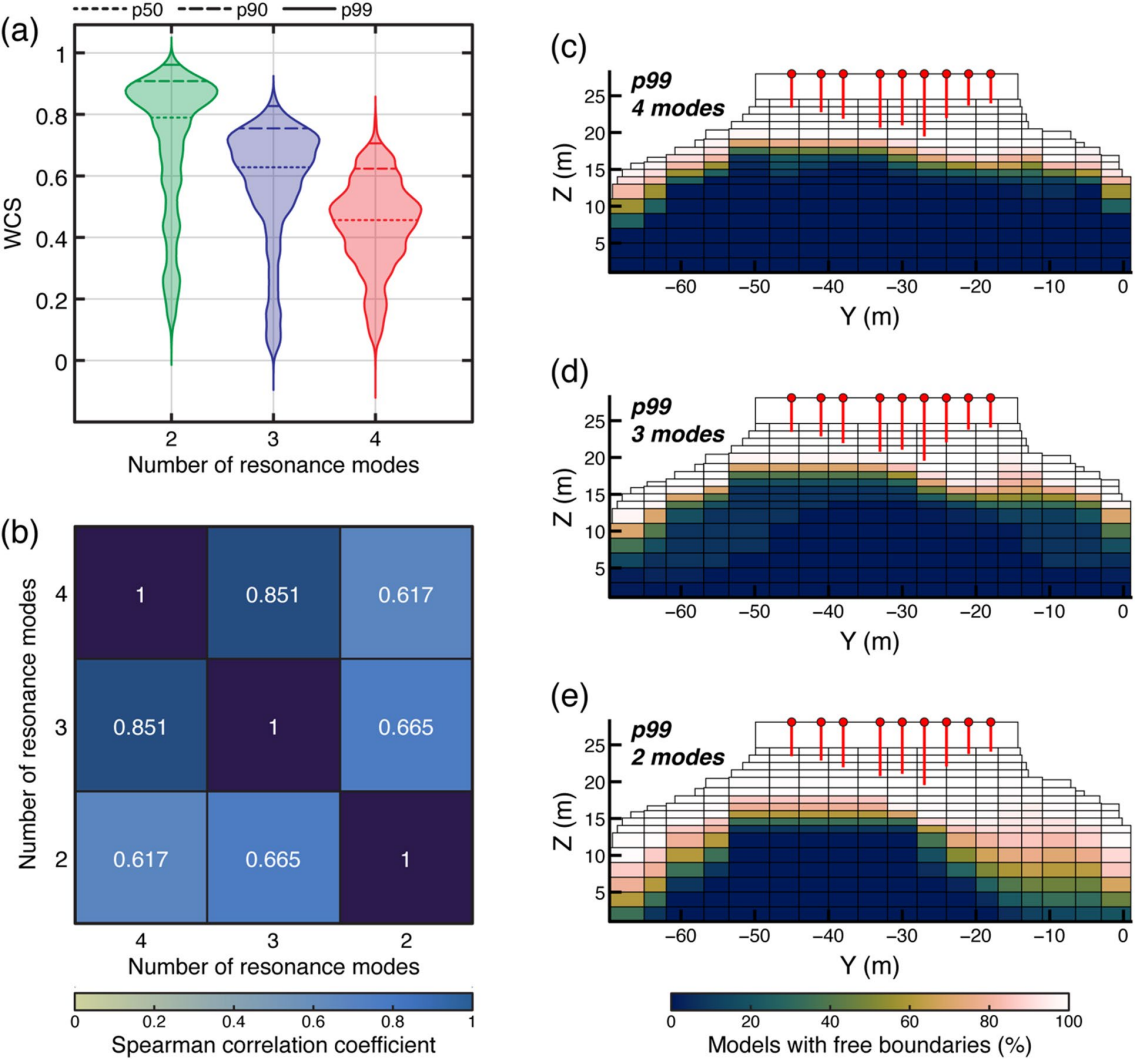
Example of the influence of resonance mode numerosity on model discrimination performance at Paradise Bay. **(a)** Distribution of WCS for including two, three, and four resonance modes in the analysis. The progressive shift and broadening of the distributions suggest score inflation and reduced discriminative potential as experimental constraints are decreased. **(b)** Spearman rank correlation matrix quantifying the stability of model rankings. The decrease in correlation coefficients indicates increased ranking instability as fewer modes are considered. **c–e)** Probabilistic fracture-persistence maps derived from the top 1% (p99) model subsets for the three analysis setups. All maps represent boundary distributions consistent with field measurements, but considering fewer resonance modes results in a broader spatial spread of probability fields, leading to less reliable constraints on the inferred fracture geometry.

Furthermore, field data quality and model representativeness also heavily constrain the performance of our approach. Accurate reconstruction of mode shapes depends on the density and geometry of seismic arrays, and sparse or uneven deployments can reduce the ability to resolve complex modes^30,46,61^. Since the slope instability geometry must be predefined, or at least hypothesized, ambient vibration measurements serve a twofold purpose: providing an experimental basis for model calibration and constraining the geometry of the slope instability when boundary conditions are obscured^31,51,65^. Likewise, the number of detectable resonance modes directly influences the stability of the ranking metrics, as shown through a sensitivity analysis of the Paradise Bay site, where the number of resonance modes in the WCS computation was progressively decreased (Fig. 11). Test results confirm that omitting even a single mode from the analysis broadens the ensemble of admissible configurations and therefore reduces the model’s discriminatory ability. In fact, reducing the number of resonance modes introduces an upward bias in WCS distributions, as fewer constraints allow more models to satisfy the similarity criteria (Fig. 11a). This apparent improvement reflects a loss of discriminatory power rather than improved model performance. To assess how this reduction affects the internal consistency of our results, we quantified the stability of model rankings using the Spearman correlation coefficient. This nonparametric metric evaluates the monotonic agreement in the ranks of model performance across analyses with different numbers of modes. High coefficients indicate that the same models remain top-ranked, while low values denote rank inversion and increasing inconsistency. The results show a marked decrease in ranking stability when fewer resonance modes are analyzed (Fig. 11b), as observed in the corresponding probabilistic fracture maps for the top 1% models (Fig. 11c–e). The resulting geometries broadly agree with field observations; however, their spatial coherence decreases as fewer modes are included, revealing increasing uncertainty in reconstructed subsurface boundary conditions. The reliability of our numerical inversion approach must be evaluated on a case-by-case basis, accounting for site-specific geological and structural complexities. In particular, seismic array deployments should be designed to comprehensively characterize the dynamic behavior of the unstable volumes, rather than following a prescribed array-density criterion. Similarly, while considering multiple resonance modes enhances the stability and selectivity of the inversion, the number of reliable modes is constrained by site conditions and data quality. In this sense, our results illustrate general trends rather than prescriptive thresholds, emphasizing the need for careful, site-specific assessment when applying the proposed approach.

While our approach was developed for toppling instabilities, its conceptual structure can be generalized to other failure mechanisms, provided that the governing kinematics and boundary conditions are appropriately characterized. However, different failure mechanisms (e.g., planar sliding or wedge failure) involve distinct fracture geometries and compliance, which would require alternative scenario-generation rules and additional parameters to characterize their mechanical behavior. Moreover, the applicability of modal analysis-based inversion depends on the presence of clear resonance modes, which are most readily observed when rock structures are sufficiently decoupled to vibrate freely. For these reasons, transfer to other failure mechanisms is feasible at a conceptual level but requires dedicated and careful development.

Despite these limitations, our inversion approach improves and extends the applicability of ambient vibration modal analysis for landslide hazard assessment. Field and numerical modal analyses have already proved effective in refining geostructural models of unstable slopes, constraining their seismic response and supporting long-term structural health monitoring^18,26,28,64^. In this framework, conducting repeated surveys—or even monitoring ambient vibrations through permanent array deployments—can help identify reversible changes in resonance modes caused by environmental factors (such as temperature fluctuations and rainfalls) or detect permanent structural damage^28,66^. Typically, such variations are analyzed only in terms of resonance frequency drifts, whereas changes in the associated mode shapes are rarely examined, especially for higher-order resonance modes^30^. However, in fracture-controlled systems, mode shapes may provide increasingly detailed insights into fracture response to external stresses and damage accumulation. By inverting these changes in modal characteristics, it may be possible to link resonance shifts to localized variations in contact stiffness, eventually describing the spatial propagation of fractures. Ultimately, the possibility of retrieving detailed descriptions of subsurface fracture configurations will expand traditional frequency-based structural health monitoring approaches, enabling refined identification of areas where structural damage clusters and develops.

## Conclusion

In this study, we attempted to transform a highly under-constrained inversion problem—the reconstruction of fracture geometries from ambient vibration data—into a structured and testable framework grounded in admissible geological and mechanical assumptions. By embedding geological realism into the stochastic generation of unique fracture scenarios and quantitatively evaluating agreement between numerical and field results through multi-metric comparison, we provide a reproducible strategy for calibrating boundary conditions of fracture-controlled rock slope instabilities. This approach aims to bridge the gap between field observations and numerical modelling, enabling modal analysis results to inform reconstruction of fracture persistence and connectivity at depth. Our results demonstrate that integrating ambient vibration modal analysis with numerical modelling provides a powerful means to investigate obscured geometry and boundary conditions of fractured rock slopes. By systematically exploring thousands of fracture scenarios and ranking consistency between simulated and measured modal parameters, we established a quantitative link between dynamic behavior and the degree of structural coupling for toppling rock slabs. One of the main advantages of our approach lies in its ability to inform boundary conditions beyond the reach of direct observation, complementing geological and geotechnical investigations with a non-invasive and replicable analytical framework.

Beyond specific application to the two investigated sites, the method we developed may establish a generalizable framework for the dynamic and structural characterization of various natural rock landforms. Our approach enables systematic exploration of boundary-condition uncertainty, guiding the identification of boundary configurations that are both consistent with measured dynamic behavior and geologically realistic. The consistency between measured and modelled mode shapes confirms that resonance modes are sensitive indicators of fracture control on slope instabilities, turning ambient vibration data from descriptive observations into diagnostic tools for subsurface characterization. Ultimately, our results may contribute to advancing the use of ambient vibration analysis for structural health monitoring and stability assessment of unstable rock slopes featuring different failure mechanisms. By coupling physically informed model generation with quantitative validation metrics, the discriminatory power of passive seismic methods is enhanced, supporting the development of scalable, non-invasive monitoring strategies for fractured rock masses in both natural and engineered environments. Future developments could focus on coupling this inversion framework with continuous monitoring applications to track temporal variations in modal properties—an essential step toward predictive, vibration-based structural health assessment of unstable rock slopes.

## Data Availability

Seismic data for Courthouse Mesa used in this study are available at 10.7914/SN/5P_2013[^67^]. Seismic data for Paradise Bay generated in this study are available at 10.5281/zenodo.17590913 [^68^]. 3D models, COMSOL Multiphysics setup files, numerical modeling results, and MATLAB codes used for fracture-scenario generation and plotting are available at 10.5281/zenodo.17600060 [^69^]. All figures were prepared using perceptually uniform and color-vision deficiency friendly colormaps available at 10.5281/zenodo.1243862 [^70^].
